# When (if ever) may doctors discuss religion with their patients?

**DOI:** 10.1111/bioe.13111

**Published:** 2022-11-20

**Authors:** Lauren Notini, Justin Oakley

**Affiliations:** ^1^ Monash Bioethics Centre, School of Philosophical, Historical and International Studies Monash University Melbourne Victoria Australia; ^2^ Melbourne Law School University of Melbourne Melbourne Victoria Australia; ^3^ Biomedical Ethics Research Group Murdoch Children's Research Institute Melbourne Victoria Australia

**Keywords:** chaplain, doctor–patient relationship, goals of medicine, professional roles, religion, social remit

## Abstract

There is ongoing debate within the bioethics literature regarding to what extent (if any) it is ethically justifiable for doctors to engage in religious discussion with their patients, in cases where patients cite religious considerations as influencing their medical decision‐making. In this paper, we concede that certain forms of religious discussion between doctors and patients are morally permissible (though not necessarily morally obligatory), insofar as patients’ religious beliefs may comprise an important part of their overall wellbeing and can influence their medical decisions. However, we argue that it is not morally permissible for doctors to engage in *substantive* religious discussion with their patients, beyond simply inquiring about the patient's values (which may include their religious values) or referring patients to a chaplain or religious figure for further discussion. In support of this claim, we put forward two key arguments which have remained relatively unaddressed in the current debate. First, we argue that it is not practical for doctors to engage in substantive religious discussion with patients, and hence it cannot be morally obligatory for them to do so. Second, we argue that, while doctors might have a professional duty to ensure that their patient's religious interests (if any) are addressed, this does not entail that *doctors* themselves are the ones who should directly address these interests. Along the way, we anticipate and respond to some possible objections to these two key arguments.

## 1 INTRODUCTION

There is ongoing debate within the bioethics literature regarding whether, and to what extent, it is ethically justifiable for doctors to engage in religious discussion with patients, in cases where patients identify as religious and cite religious considerations as influencing their medical decision‐making.^1^ Some argue that doctors have a moral duty to engage in substantive religious discussions with religious patients in certain circumstances. For example, Kuczewski argues that, given doctors’ informed consent obligations and the importance of good caregiver–patient relationships, ‘in some more recalcitrant conflicts regarding treatment plans, resolution may require that clinicians become more involved and personally engage in discussion and disclosure of religious and spiritual worldviews’.^2^ Similarly, Brett and Jersild argue that it is sometimes ‘appropriate for clinicians to explore the theological basis of a family's decisions and to offer alternative [religious] interpretations…Clinicians should not simply acquiesce to religious reasoning when they are asked to provide medical care they believe is medically inappropriate or inhumane. Rather, clinicians should continue active discussion…[and] in selected cases, it is appropriate for clinicians to advance alternative theological arguments’.^3^ And Pellegrino and Thomasma argue that ‘If they share these [religious] beliefs, the physician can then help the patient to draw upon the spiritual resources available within him or herself, the community of believers, and the faith in order to make authentic decisions’.^4^ However, others argue that doctors cannot justifiably engage in religious discussion with patients, since doctors lack the professional competencies required to effectively do so, the public nature of doctors’ role limits their appealing to public/secular rather than religious considerations, and doctors who engage in such discussion may undermine patients’ trust (if patients expect their doctors to focus on their medical interests, only) and damage the doctor–patient relationship.^5^

It is important to clarify at the outset that there are different types and degrees of religious discussions. At one end of the spectrum, a doctor might simply inquire about whether a patient is religious, as part of taking a holistic patient history or clarifying the values influencing the patient's medical decision‐making. At the other end of the spectrum, a doctor might engage in more substantive religious discussion, for example, by attempting to highlight errors in patients’ religious reasoning, or challenging patients’ interpretation and application of certain religious tenets when making medical decisions.^6^ We focus on instances where the patient's religion is influencing their medical decision/s, and on more *substantive* religious discussions—that is, religious discussion which goes beyond merely inquiring about patients’ religious values, or merely referring patients to a religious figure, chaplain or another member of the hospital's pastoral care team. This definition of ‘substantive religious discussion’ is intended to be quite broad, and does not include activities which seem to uncontroversially fall within the scope of doctors’ professional remit as doctors (e.g., referring religious patients to chaplains). What we have in mind by such discussions includes activities such as doctors attempting to correct what they regard as errors in patients’ religious reasoning from metaphysical premises about miracles or about the nature of God's existence; doctors engaging more directly with the normative grounds for patients’ religious reasoning (by maintaining ‘that the stated normative reason…does not apply in the manner the patient….assumes…or that it should be reinterpreted’);^7^ and doctors seeking to acquire a deep and shared understanding of patients’ religious values and how these are influencing their medical decisions. So, both the nature and extent of the discussion may determine how ‘substantive’ the religious discussion is.^8^

We focus only on religious discussion between doctors and patients, rather than religious discussion between doctors and others (e.g., other doctors). This is because the nature of the doctor–patient relationship is fundamentally different to the nature of other relationships doctors may have (e.g., with colleagues). Unique features of the doctor–patient relationship include a power imbalance (e.g., some patients may automatically defer to their doctors), trust, and doctors’ professional duty to promote their patients’ wellbeing, using the proper tools of medicine and within the scope of their professional role. Our arguments are intended to apply both in situations where doctors work as isolated individuals (e.g., a rural general practitioner) as well as where doctors work as members of an interprofessional team (e.g., in a hospital setting or multidoctor general practice).

We concede that certain forms of religious discussion between doctors and patients are morally permissible (though not morally obligatory), insofar as patients’ religious beliefs may play an important role in their overall wellbeing and can influence their medical decisions. However, we argue that it is not plausibly held to be morally obligatory, or morally permissible, for doctors to engage in *substantive* religious discussion with their patients, beyond simply inquiring about patients’ values (including any religious values), or referring patients to a chaplain or religious figure for further discussion. In support of these claims, we put forward two key arguments which have remained relatively unaddressed in the current debate. First, we argue that it is not practical for doctors to engage in substantive religious discussion with patients, and hence it cannot be morally obligatory for them to do so (the *Impracticality Argument*). Second, we argue that, while doctors might have a professional duty to ensure that their patient's religious interests (if any) are addressed, this does not entail that it is *doctors* themselves who should directly address these interests (the *Professional Role Argument)*. Also, we anticipate and respond to some objections to these two key arguments.

The Impracticality Argument and the Professional Role Argument are intended to function as two separate, distinct arguments. Hence, the success (or otherwise) of our Impracticality Argument should have no bearing on that of our Professional Role argument, and vice versa. The Impracticality Argument helps to support our claim that it is not morally *obligatory* for doctors to engage in substantive religious discussion with their patients. The Professional Role argument helps to support our claim that it is not morally *permissible* for doctors to engage in this activity (even in circumstances where it might be thought practical for them to do so). Our Impracticality Argument is not intended to establish what should be considered professional behaviour by doctors. Similarly, our Professional Role argument is not intended to establish what it is practical for doctors to do.

## 2 THE IMPRACTICALITY ARGUMENT

An implicit assumption in claims that doctors are sometimes morally obligated to engage in substantive religious discussion with their patients is that, it is practical for doctors to do this. In this section, we challenge this assumption, and put forward some key reasons as to why this practice may be impractical. As it is commonly thought that ‘ought implies can’, our Impracticality Argument helps to support our claim that it is not plausible to hold that doctors are morally obligated to engage in this activity.

Some authors who argue that it is not morally permissible (and so, *a fortiori*, cannot be morally obligatory) for doctors to engage in substantive religious discussion with their patients cite a lack of the requisite competencies as a key reason for this. (We refer to this as the ‘lack of professional competencies’ argument). For example, Schuklenk claims:

Doctors, *in their role as doctors*, have no *professional* competencies^9^ when it comes to religion…If there are patients who seek spiritual guidance from their doctor, it would be important for the healthcare professional to flag precisely their lack of professional competency vis‐à‐vis that particular patient request/need and suggest they seek advice on spiritual matters from a third party capable of delivering that advice. That is so, even in [the] case of particular doctors who happen to share the religious or other ideological preferences of (some of) their patients. That shared preference does not render them experts on that subject matter.^10^

Importantly, this argument concerns a lack of *professional* competencies, rather than a lack of competencies (in some unspecified way). It is possible that some doctors may indeed be (or at least perceive themselves to be) competent, as *individuals*, to engage in substantive religious discussion with their patients. This may include doctors who have theological,^11^ in addition to medical, training, or doctors who are familiar with the patient's religion (e.g., where the doctor and patient follow the same branch of the same religion^12^). However, this does not necessarily mean that doctors are *professionally* competent (as doctors, by virtue of their professional role) to engage in substantive religious discussion with their patients. Thus, to highlight how some individual doctors might have sufficient religious understanding and competence to engage in substantive religious discussions with their patients would be beside the point of our argument here, which is about what is plausibly viewed as morally required of anyone who occupies the role of doctor.

There is empirical evidence to support the ‘lack of professional competencies’ argument. Best and colleagues conducted a systematic literature review of 61 empirical studies, which collectively included reports from over 20,044 doctors.^13^ This review found that, while most doctors regard spiritual/religious wellbeing as an important component of health, in most contexts doctors discuss religion and spirituality only infrequently with their patients and generally prefer to refer patients to chaplains for such discussion. Doctors reported discussing religion and spirituality with patients in end‐of‐life contexts (where patients may have greater spiritual needs) somewhat more frequently than in other contexts, although a number of the studies reviewed found that such discussions were still somewhat infrequent even in end‐of‐life contexts. Some of the studies reviewed found that 2% to 46% (median 18%) of doctors reported that they would never discuss religion and/or spirituality with their patients, including in cases where patients directly asked for this, and/or were at the end of their lives.^14^ Where religion and/or spirituality were raised by patients, most doctors reported attempting to change the subject.^15^ Doctors commonly cited lack of training and/or insufficient knowledge as reasons why they avoid discussing religion and/or spirituality with their patients. Doctors who had received religion/spirituality training in the clinical context^16^ and/or were more religious/spiritual were the most likely to have such discussions with, and provide spiritual care to, their patients.^17^ This finding held regardless of the patient's and doctor's religion. However, some doctors reported that they avoid discussing religion/spirituality with patients who held different religious/spiritual beliefs to themselves.^18^

Interestingly, Rasinksi and colleagues found the *source* of any religious or spirituality training to be relevant to whether doctors discussed religious/spiritual matters with patients. They found that doctors who had received religion and spirituality training from continuing medical education, books, or through their own religion, more often discussed religious/spiritual matters with patients, compared to doctors who had received such training during medical school.^19^ This suggests that, even if religion/spirituality training *were* routinely included in medical school curricula (as some may argue it should be), this alone may be insufficient to increase the frequency with which doctors discuss religious/spiritual matters with patients. Furthermore, while the routine inclusion of religion/spirituality training in medical schools may make doctors more likely to discuss religion/spirituality with patients, this also raises practical issues. Doctors would need to be taught to understand different religions that they may encounter in patient consultations. This may be sufficient for doctors to feel comfortable with briefly discussing their patient's religion, in cases where patients raise this during the medical consultation (e.g., to better understand the values motivating the patient's medical decision). However, this basic level of training may be insufficient to enable doctors to engage more deeply in religious discussion, such as highlighting errors in patient's religious reasoning (an activity some^20^ argue doctors should engage in), which would require more advanced theological understanding. To partake in such discussion, doctors would need to not only have an in‐depth understanding of each different religion but also of different versions of the same religion. Such understanding would take significant time to cultivate; and the time required to acquire such understanding would result in less time being available for the strictly ‘medical’ aspects of training and/or would necessitate extending the duration of professional training. Alternatively, this training could be completed as part of continuing professional development. However, as not all doctors would be available for and/or interested in undertaking this additional training, any expectation that all doctors would have acquired a sufficiently in‐depth understanding of various religions to correctly highlight any ‘objective’ errors in patients’ religious reasoning may be unrealistic.

Perhaps a compromise argument could be reached; it could be argued that *if* doctors have sufficient competence to engage in substantive religious discussion with their religious patients, then it is plausible to hold that they can in certain circumstances be morally obligated to do so. This argument would obligate the presumably small subset of doctors who are competent in engaging in substantive religious discussion to do so with their religious patients, without expecting or requiring *all* doctors to do this. However, this may also raise concerns relating to lack of role clarity, dual roles, and/or potential conflicts of interest. For example, patients (and the broader community) may question what role a doctor is fulfilling (e.g., doctor and/or religious advisor) if the doctor engages in substantive religious discussion with their patients. Conflicts of interest may arise where engaging in such discussion means that doctors allow another interest (e.g., getting the patient to agree with their religious perspective) to override their primary duty to act in their patient's best (medical) interests.

It might also be suggested that, while it is not practical for a single doctor to be well‐versed in all religions (and their different branches), it may be possible for teams of doctors to have the requisite expertise. For example, one doctor working within a hospital or general practice team may have expertise in one religion, while their colleague may have expertise in another.^21^ Importantly, such expertise would be *personal*, rather than *professional*, expertise, given the nature of doctors’ professional boundaries (described in the next section). One would also need to account for idiosyncrasies in how different individuals interpret the same branch of the same religion; a doctor and patient who share the same religion may have very different religious understandings and interpretations.

One might also question how widely applicable our Impracticality Argument is. For example, it could be claimed that our Impracticality Argument may not apply in certain contexts (even if it were deemed sound in other contexts), such as in communities with many religious doctors knowledgeable regarding their and their neighbours’ faith. Similarly, the strength of our Impracticality Argument over time could also be questioned (e.g., if the number of religious doctors increased over time).^22^ However, in such situations, it will still be important to account for idiosyncrasies in the practise of religion; a doctor and patient, even if they share the same faith, may have very different understandings of the same religious directive and how it ought (not) to be applied to a medical decision. In addition, even if it were more practical for doctors in certain contexts to engage in substantive religious discussion with their patients, this does not entail that doctors *should* be doing so, given professional boundary concerns.

Resource allocation issues present another reason why it may not be practical (or just) for doctors (even those who possess the requisite competencies) to engage in substantive religious discussion with their patients. If doctors take the time to engage in such discussion with their patients, they will have less time to attend to their patients’ medical needs, at least to the extent that these are separate from patients’ religious/spiritual needs. Best and colleagues’ systematic review identified insufficient time as the most common barrier to asking patients about their religion/spirituality, as cited by doctors.^23^ Doctors’ time is a limited resource. Time spent by doctors engaging in substantive religious discussion with their patients is time that could be spent elsewhere. Engaging in such discussion with a patient who seeks this could be carried out by a different professional (e.g., a chaplain). However, only doctors can attend to patients’ medical needs.

It might also be suggested that religion/spirituality training in medical education need not be particularly arduous. Such training may not be about identifying errors in patients’ religious reasoning but rather simply ensuring that doctors have sufficient knowledge to enable them to engage intelligently and understand patients’ religious reasoning to demonstrate that patients have been understood, and for doctors to compassionately assist their patients with making informed medical decisions.^24^ However, for reasons noted earlier, another individual (e.g., a chaplain) could do this same work (and likely more effectively). Furthermore, delegating this to another individual (e.g., a chaplain) would mean that this same goal can be achieved but by someone for whom this activity falls within their professional remit, as we argue (more fully) in the next section.

## 3 THE PROFESSIONAL ROLE ARGUMENT

We have argued so far, using our Impracticality Argument, that it is implausible to hold that it is *morally obligatory* for doctors to engage in substantive religious discussions with religious patients (at least, in the circumstances mentioned by those who make this claim). However, this critique leaves it open whether it might still be *morally permissible* for doctors to engage in substantive religious discussions with religious patients in such circumstances, where a doctor can feasibly do so—because, for instance, they have the requisite training, time, and resources. Suppose the various impracticalities we highlighted above (e.g., insufficient training and resources for doctors to engage in substantive religious discussions) were addressed and overcome. Would it then be plausible to claim that it can be at least *morally permissible* for doctors to engage in substantive religious discussions with (religious) patients? In this section we argue that this claim is not plausible.

One could invoke the is/ought distinction to highlight that, while the available empirical evidence showing that doctors appear to receive no or little training in religion as part of their medical education (and therefore often feel incompetent to effectively discuss religion and spirituality with their patients) tells us what *is* the case, it does not tell us what *ought* to be the case. Perhaps it would be beneficial and valuable for doctors (and therefore for patients) to receive such training as part of routine medical education.^25^ Nevertheless, this would not entail that it is morally permissible (still less that it is morally obligatory) for *doctors* themselves to directly address those interests. Our Professional Role argument, advanced in this section, is intended to help answer the normative question of what *should* occur in practice.

One reason for holding that it is not morally permissible for any religious interests of patients to be substantively addressed by doctors is that doing so might plausibly be thought to involve doctors going beyond the proper boundaries of their professional role, in ways which would unacceptably breach the scope of their social remit (i.e., what society has authorised them to do, as doctors). Thus, for example, Robert Baker has highlighted, during the secularisation of the medical profession in America in the mid‐19^th^ century, doctors who combined religious ethics or religious beliefs with medical decision‐making in patient care came to be regarded as ‘unprofessional’.^26^ Baker illustrates the boundaries of doctors’ professional roles in part by referring to codes of ethics issued by professional medical associations, such as the American Medical Association (AMA). As Baker explains, in developing its pioneering code of medical ethics during the 19th century,^27^ the AMA explicitly sought to separate doctors’ professional role from religion and religious beliefs.

We agree that doctors’ professional role does not extend to engaging in religious discussions with religious patients, and that the statements and codes of ethics of professional medical associations can help delineate the boundaries of doctors’ professional obligations. But while Baker himself does not define doctors’ professional role solely in terms of those which are outlined in such statements and codes,^28^ there would in any case be significant limitations with relying solely on such statements and codes in determining doctors’ professional role boundaries and the scope of their social remit. As Goldman argues, ‘Professionals cannot just arbitrarily adopt their own moral rules; their special positions spelled out in their codes of ethics must be accepted by society or the state and therefore be perceived as expressing priorities that all should be willing to accept’.^29^

To reinforce this point about doctors’ professional roles embodying a *social* remit, consider the monopoly of expertise argument for the scope of a professional's role and their role obligations and role virtues. That is, members of a profession are granted a monopoly of expertise on the provision of key goods, which other members of society are not trained or authorised to provide. It is commonly (and plausibly) thought that, in return for being granted this monopoly, members of a profession have an obligation to make their services *broadly available* to the community, and so to not pick and choose their clients or patients based on the practitioner's personal preferences. Thus, doctors owe the community a readiness to provide the requisite medical services to enable patients to attain and maintain the key good of health, and the community justifiably expects *any* occupant of the role of doctor to provide such services. However, doctors have not been granted a monopoly of expertise on the provision of religious advice to patients, and the provision of such advice is not something which the community can justifiably expect of each occupant of the role of doctor. Even if some doctors might also happen to have considerable religious expertise, it is not appropriate for doctors, *qua* doctors, to hold themselves out to the community (and to be regarded by patients) as being experts on religious matters. After all, in what capacity would a doctor be acting, if they gave religious advice to patients? These considerations are grounds for thinking that those who advocate incorporating the provision of religious advice to patients as a mandatory (or at least morally permissible) part of doctors’ professional role must meet a heavy burden of proof to succeed in demonstrating that providing such advice should be added to doctors’ social remit.

Dare highlights the importance of practitioners fulfilling and remaining within their social remit, in his discussion of the professional role of lawyers:

Few people manage to get through their lives without requiring the services of a professional…it is important not only that professionals are ethical, but that clients and potential clients have some way of knowing the ethical stance of practitioners even though they do not know them or their moral views personally. The adoption and promulgation of a distinct professional morality is a way of making the ethics of the profession public in a way that the personal ethics of its members cannot be. Clients get the benefit of these ‘public ethics’ however, only if they are indeed given priority over personal ethics in members’ dealings with the public. The client need only know what values the professional role requires the professional to adopt and that the professional is a role‐occupant, to know what values at least should govern the professional's conduct in the relationship.^30^

Role clarity is important, as it is crucial to be clear on which individuals are fulfilling which roles in healthcare, and which ‘hat’ an individual is wearing at any one time (their professional ‘hat’ or their personal ‘hat’). Lack of role clarity may lead to confusion, different individuals doubling up on the same role (and therefore inefficient use of resources), or one individual fulfilling a role that could be more appropriately fulfilled by another.

Important moral bases for such professional codes and statements are deeper considerations about the nature of medicine, along with the fundamental moral commitments doctors take on when they join the profession. After all, professional codes can change significantly over time, and some of those changes are widely seen as improvements constituting moral progress. For example, medical codes of ethics are now generally far less paternalistic towards patients than those codes used to be. And yet, for such claims of moral progress to be coherent, there must be some underlying basis by which earlier and later codes of medical ethics can be compared. Indeed, were codes of ethics to be the fundamental basis of doctors’ professional commitments, this would implausibly suggest that, in circumstances where any such code is absent, doctors working in that area have no professional duties at all. So, a deeper grounding of the scope of doctors’ professional role and social remit is needed, which goes beyond (and might sometimes even conflict with) the statements and precepts in a particular code of medical ethics, to clarify doctors’ social remit.

We believe that doctors’ professional role and their social remit as doctors, and what defines the practice of medicine, (and hence our Professional Role argument) do not simply relate to the tools of medicine, but rather are more plausibly based in an account of the proper goals of medicine.^31^ And indeed, this approach can provide an attractive justification for claims about changes in codes of ethics constituting moral progress, as this approach could view one particular code of medical ethics as morally better than another when the former code enables doctors to serve the proper goals of medicine better than does the latter code. One version of such an approach highlights links between the proper goal/s of the profession in question (e.g., health for medicine) and an Aristotelian conception of human flourishing (*eudaimonia*). This proper goal/s can, in turn, provide the basis for an account of the professional role obligations, and also of the role virtues, for that profession.^32^ This social remit for the profession also arguably includes a core practitioner responsibility to remain alert to, and to highlight, when certain professional practices and particular directives in the relevant professional codes (even if widely followed) evidently fail to serve well the proper goals of the profession.

Some might argue that the proper goal of medicine should be construed more broadly, as the promotion of overall patient wellbeing, including the patient's physical, mental, and social wellbeing, and not only as the promotion of the patient's physical health.^33^ Moreover, some may argue that the patient's religion (if any) is an important part of their overall wellbeing (which we would not dispute) and that therefore *doctors* can permissibly address this.

However, in response to this, it seems plausible to suggest that doctors can still help in *indirect* ways with ensuring that their patient's religious interests (if any) are addressed, for example, by referring the patient to a chaplain and/or religious figure to consult with. After all, doctors do not have a duty to do anything and everything to promote their patients’ wellbeing. Consider a patient who is homeless. Their wellbeing would undoubtedly be significantly promoted if they were gifted a house. But this does not entail that the doctor is obliged to buy this patient a house. Doctors’ social remit relates to their using the *tools of medicine* (e.g., medical training, diagnosis, medication, surgery/procedures, and equipment) to promote patient wellbeing. Perhaps the doctor has a weak obligation to connect the patient with housing services (since the need for housing is a basic need and should be addressed). But then the same could be argued in the case of religion—that is, the doctor has a more modest obligation to refer the patient to a chaplain (providing the patient consents to this) or religious figure or service of the patient's choice. In these respects, chaplains could be regarded as another type of specialist that a doctor might appropriately refer their patients to, when the relevant needs exist.

A concern about unjust discrimination might be raised against our arguments so far. That is, some might respond to our Impracticality Argument by suggesting that it is also impractical for doctors to engage in substantive discussion about patients’ *nonreligious* beliefs, insofar as it is challenging for doctors to understand and engage with these beliefs, and doing so may also mean that doctors have less time to engage in strictly ‘medical’ work. Hence, permitting doctors to engage in substantive discussion with patients about patients’ nonreligious beliefs, but not their religious beliefs, could be thought to involve unjust discrimination.^34^ We agree that, as there might be no inherent difference between religious and other types of personal beliefs, and that nonreligious beliefs are not necessarily easier to understand and engage with than religious beliefs, they should therefore be treated similarly. However, this similarity does not entail that doctors should therefore be permitted (still less, morally obliged) to engage in substantive discussion with patients about patients’ religious beliefs. Religious and nonreligious beliefs can be treated equally, but the opposite conclusion could be reached: that doctors should not substantively engage with either patients’ religious or nonreligious personal beliefs, as this is impractical and not within the scope of doctors’ professional remit. This is not to say that patients’ beliefs—religious or nonreligious—should not be engaged with by *anyone*. Indeed, such beliefs often have important implications for decision‐making (including medical decision‐making) and would therefore deserve serious engagement. For example, a patient may have a strong personal belief that their organs should be donated only to people of their race because they believe that this group has previously been subjected to unjust organ donation practices.^35^ This belief would have important implications for the patient's transplant decisions and should be thoroughly unpacked and explored (perhaps by a psychologist or social worker). However, for reasons relating to impracticality and professional role boundaries, *doctors* should not be the group of individuals who do this work of engagement.

What about doctors who refuse to refer patients elsewhere for substantive religious discussion? One could draw similarities with doctors’ referrals in other contexts, such as abortion.^36^ For example, some authors and professional organisations have argued that, while doctors have the right to refuse to perform abortions, they have a duty to refer the patient to a willing doctor.^37^ However, others have argued that doctors have no such duty to refer, as referring may make them morally complicit.^38^ It is also important to highlight that the reason for the doctor's refusal matters. If the doctor's refusal to provide an abortion is personal/religious in nature, this is considered conscientious objection.^39^ Such a doctor may claim that while *they* cannot perform an abortion, it is acceptable for a different doctor to do so (and such a doctor may not believe that referring would make them morally complicit). Other doctors who refuse to perform abortions may have professional reasons for doing so; the idea that *no* doctor should perform abortions. This type of refusal would constitute a professional integrity/role objection, rather than a conscientious objection. Hence, a doctor who refuses to perform an abortion for these professionalism‐based reasons may also object to referring the patient to a willing doctor on such grounds. While it is plausible that a doctor may object to engaging in substantive religious discussion with their patients for moral/personal reasons (e.g., a dislike of or lack of interest in religion), it seems more likely that doctors’ refusals to engage in such activity would be grounded in professionalism‐based reasons (e.g., a belief that doctors who engage in this activity would be acting beyond their professional boundaries as doctors). If this is true, then it is likely that such doctors would (and should, according to our Professional Role argument) also object to referring patients to *other* doctors for substantive religious discussion (even to doctors who are willing and feel qualified to engage in such discussion). However, if one maintains that it is professionally appropriate for others (e.g., chaplains or religious figures/services) to engage in substantive religious discussion with patients, then a professionalism‐based reason would not legitimately underpin a doctor's refusal to refer patients to these sources. While we acknowledge the possibility that referring patients to chaplains or religious figures/services may be a moral obligation (rather than merely permissible), more philosophical work is needed to establish whether such referral by doctors is, in fact, morally obligatory, and such work is beyond the scope of this paper.

A concern could be raised that, if doctors misunderstand how a patient's religion is relevant to the patient's decision‐making process, or delegate substantive religious discussion to chaplains or religious figures/services (because they are not adequately trained to do so, or believe this is not part of their professional role), this could have negative consequences. For example, it could inadvertently result in resources being used less effectively, lack of compassion, less‐informed clinical decision‐making, and adverse clinical outcomes. Therefore, doctors who engage in substantive religious discussion with their patients may be more resource‐savvy and achieve better patient outcomes.^40^ But that need not be the case. A doctor referring a patient to a chaplain (or religious figure/service) for substantive religious discussion need not mean that such discussion should have *no* bearing on medical discussions and decision‐making. For example, a chaplain could do the work of substantive engagement, unpacking the patient's religious values further and establishing how these are influencing the patient's medical decision‐making. The chaplain could then report back to the doctor (much as a specialist would), perhaps even translating this discussion into more secular terms. The doctor may then use this information to inform or provide context to clinical decision‐making or their communication with the patient. Patients’ beliefs (religious or nonreligious) could be justifiably considered when making other types of decisions with implications for patients (e.g., resource allocation decisions)—however, this does not entail that doctors should (or can permissibly) be the ones to substantively engage in and discuss such beliefs with patients. It is possible for patients’ voices to be sought and represented in healthcare discussions and decisions in other ways that do not transgress doctors’ professional boundaries. 41

## 4 CONCLUSION

We have argued that it is not morally obligatory, nor morally permissible, for doctors to engage in substantive religious discussion with their patients (that is, discussion which goes beyond simply inquiring about the patient's values or referring patients to a chaplain or religious figures for further discussion), where patients identify as religious and cite religious considerations as influencing their medical decision‐making. We put forward two separate arguments, the ‘Impracticality Argument’ and the ‘Professional Role Argument’, in support of this claim. While a number of authors argue that doctors can justifiably engage in substantive religious discussion with their patients, a key feature which is often overlooked in the current literature relates to the practicality of doctors doing so. Our Impracticality Argument illuminates this consideration. Doctors’ time is limited, as is the time allocated to their training. It is critical that ethical arguments consider the realities of clinical training and practice.

We do not deny that religious interests are often an important component of patients’ overall wellbeing. Therefore, under welfarist accounts of the proper goals of medicine, doctors might plausibly be thought to have a professional duty to ensure that patients’ religious interests are addressed. However, according to our Professional Role Argument, doctors would have no such duty to *directly* address patients’ religious interests, but only to refer them on to others who are better trained and professionally suited to doing so (e.g., chaplains or religious figures). Doctors should receive basic training in how to professionally and respectfully decline any patient requests to directly engage in substantive religious discussion.

An advantage of our arguments is that they apply to a range of situations, including where doctors and patients may share or diverge in their religious beliefs. Doctors can and should care about their patients’ religious interests, but any expectation for doctors to directly address these interests is impractical, and does not align with doctors’ professional roles.

## Footnotes

1 For simplicity, we acknowledge but do not address patients who identify as spiritual, but not religious.

2 Kuczewski, M. G. (2007). Talking about spirituality in the clinical setting: Can being professional require being personal? *American Journal of Bioethics*, *7*(7), 4–11, p. 5.

3 Brett, A. S., & Jersild, P. (2003). “Inappropriate” treatment near the end of life: Conflict between religious convictions and clinical judgment. *Archives of Internal Medicine, 163*(14), 1645–1649, p. 1648.

4 Pellegrino, E. D., & Thomasma, D. C. (1996). *The Christian virtues in medical practice* (p. 151). Georgetown University Press. They argue that ‘The obligation to provide such [spiritual] assistance applies to [all] physicians…humans are spiritual as well as physical entities, and they cannot be healed without attending to their spiritual needs’ (p. 67). Smith and Audi argue that ‘providers’ sharing of their own spiritual beliefs and experiences can communicate understanding to patients that may be not only appropriate but virtuous…[this] may aid in showing patients and families concern appropriate to medical care’. Smith, W. R., & Audi, R. (2021). Religious accommodation in bioethics and the practice of medicine. *Journal of Medicine and Philosophy, 46*(2), 188–218, pp. 205–206.

5 Greenblum, J., & Hubbard, R. K. (2019). Responding to religious patients: Why physicians have no business doing theology. *Journal of Medical Ethics, 45*(11), 705–710; Schuklenk, U. (2019). Professionalism eliminates religion as a proper tool for doctors rendering advice to patients. *Journal of Medical Ethics, 45*(11), 713.

6 Brett & Jersild, op. cit. note 3.

7 Greenblum & Hubbard, op. cit. note 5, pp. 705–706. Greenblum and Hubbard refer to these responses by doctors as ‘the metaphysical response’, and ‘the direct normative response’, respectively. A full specification of ‘substantive religious discussion’ is beyond the scope of this paper.

8 Individuals may vary in where they set this threshold for ‘substantive’ religious discussion.

9 While Schuklenk does not define ‘professional competencies’ in this paper, our personal correspondence with Schuklenk confirms that this statement was intended as an expression of doubt that anyone can have ‘professional’ competencies in faith/religion.

10 Schuklenk, op. cit. note 5, p. 713, emphasis added.

11 Consider a medical doctor with formal theological training. This case would raise additional issues about ‘dual roles’—this doctor would need to be clear about which ‘hat’ they are wearing at a particular time. Importantly, the term ‘dual role’ here assumes that a doctor's purely ‘clinical’ role can and should be separated from any other role they may be trying to fulfil (e.g., a theological role). It also assumes that doctors’ professionalism must not incorporate a religious role (thanks to Joshua Hordern for raising these points). While there may be overlap between patients’ religious and other (e.g., medical) interests, this does not entail that doctors’ medical and nonmedical (e.g., theological) roles can justifiably overlap in the same way, given the scope of their social and professional remit as doctors. Even if a doctor could neatly separate their nonmedical and other roles, and be clear about which role they are functioning within at any one time, society has not authorised doctors to engage in substantive religious discussion with patients. This may be due to risks that religious doctors may push their religious views onto patients, that such discussion may take away from doctors’ purely ‘medical’ work, that this activity may conflict with doctors’ primary duty to promote their patients’ medical interests, or that patients may mistrust doctors who purport to have religious expertise (as part of their doctor's role). These broader risks seem to outweigh any potential benefit individual patients may gain from engaging in substantive religious discussion with their doctor.

12 While the doctor and patient may share the same religion, they may nevertheless have different understandings (e.g., different interpretations of a religious tenet/passage). This highlights the individualistic/idiosyncratic aspect of religion and raises the question of whether it is possible to be fully ‘competent’ in religion.

13 Best, M., Butow, P., & Olver, I. (2016). Doctors discussing religion and spirituality: A systematic literature review. *Palliative Medicine*, *30*(4), 327–337.

14 Ibid.

15 Ibid.

16 The content of which was unspecified.

17 Best, M., et al., op. cit. note 13.

18 Ibid.

19 Rasinski, K. A., Kalad, Y. G., Yoon, J. D., & Curlin, F. A. (2011). An assessment of US physicians’ training in religion, spirituality, and medicine. *Medical Teacher, 33*(11), 944–945.

20 For example, Clarke, S. (2013). When they believe in miracles. *Journal of Medical Ethics, 39*(9), 582–583.

21 Thanks to Joshua Hordern for raising this possibility.

22 Thanks to Joshua Hordern for raising these points.

23 Best, M., et al., op. cit. note 13.

24 Thanks to Joshua Hordern for raising these points.

25 Thanks to Joshua Hordern for highlighting this.

26 Baker, R. (2013). *Before bioethics: A history of American medical ethics from the colonial period to the bioethics revolution* (p. 182)*.* Oxford University Press.

27 American Medical Association. (1847). *Code of ethics of the American Medical Association.* T.K. and P. G. Collins.

28 See Baker's definition of ‘medical ethics’: Baker, op. cit. note 26, pp. 3–4.

29 Goldman, A. (2018). Moral epistemology and professional codes of ethics. In A. Zimmerman, K. Jones, & M. Timmons (Eds.), *The Routledge handbook of moral epistemology* (pp. 482–492). Routledge, pp. 482–483.

30 Dare, T. (1998). “The secret courts of men's hearts”: Legal ethics and Harper Lee's *To Kill a Mockingbird.* In K. Economides (Ed.), *Ethical challenges to legal education and conduct* (p. 58). Hart Publishing. See also Kant's famous example of the conflicted pastor who has personal misgivings about certain teachings of his church, yet, Kant argues, nevertheless has an overriding role obligation to preach the official teachings of the church to his congregation (though he can permissibly criticise those church teachings outside his role as a preacher); Kant, I. (1959). What is enlightenment? In I. Kant (Ed.), ( L. W. Beck, Trans.), *Foundations of the metaphysics of mo* *rals* *and What is enlightenment* (p. 88)*.* Bobbs‐Merrill.

31 Others may adopt a different account of doctors’ professionalism and therefore reach a different conclusion about what it is professional for doctors to do.

32 Oakley, J., & Cocking, D. (2001). *Virtue ethics and professional roles*. Cambridge University Press.

33 World Health Organization. (2006). *Constitution of the World Health Organization* (p. 1). http://www.who.int/governance/eb/who_constitution_en.pdf

34 Thanks to Joshua Hordern for raising this concern.

35 Thanks to Joshua Hordern for this example.

36 Thanks to Joshua Hordern for raising this potential analogy.

37 American College of Obstetricians and Gynecologists. (2007). The limits of conscientious refusal in reproductive medicine: ACOG committee opinion no. 385. *Obstetrics & Gynecology, 110*(5), 1203–1208.

38 Bayles, M. D. (1979). A problem of clean hands: Refusal to provide professional services. *Social Theory and Practice*, *5*(2), 165–181.

39 Fiala, C., & Arthur, J. H. (2017). There is no defence for ‘Conscientious objection’ in reproductive health care. *European Journal of Obstetrics & Gynecology and Reproductive Biology*, *216*, 254–258.

40 Thanks to Joshua Hordern for raising these points.

41 For example, by a patient representative on a hospital committee, or a chaplain reporting on the patient's religious views at a multidisciplinary team meeting.

